# Severe dynamic left-ventricular outflow-tract obstruction unmasked by transcatheter aortic valve implantation: a case report

**DOI:** 10.1093/ehjcr/ytag755

**Published:** 2026-10-14

**Authors:** Zongmei Gao, Minghui Yuan, Yi Li, Jingli Lu, Ruijian Li

**Affiliations:** Department of Cardiology, Affiliated Hospital of Shandong University of Traditional Chinese Medicine, No. 42 Wenhua West Road Jinan, 250011 Shandong, People's Republic of China; Department of Cardiology, Affiliated Hospital of Shandong University of Traditional Chinese Medicine, No. 42 Wenhua West Road Jinan, 250011 Shandong, People's Republic of China; Department of Cardiology, Affiliated Hospital of Shandong University of Traditional Chinese Medicine, No. 42 Wenhua West Road Jinan, 250011 Shandong, People's Republic of China; Department of Cardiology, Affiliated Hospital of Shandong University of Traditional Chinese Medicine, No. 42 Wenhua West Road Jinan, 250011 Shandong, People's Republic of China; Department of Cardiology, Affiliated Hospital of Shandong University of Traditional Chinese Medicine, No. 42 Wenhua West Road Jinan, 250011 Shandong, People's Republic of China

**Keywords:** Aortic stenosis, Transcatheter aortic valve implantation, Dynamic outflow obstruction, Systolic anterior motion, Case report

## Abstract

**Background:**

Relieving the fixed afterload of severe aortic stenosis can unmask a previously concealed dynamic outflow-tract obstruction.

**Case summary:**

A 69-year-old man with severe aortic stenosis (valve area 0.65 cm^2^; 109/64 mmHg), mild concentric hypertrophy (septum 15 mm), and no baseline systolic anterior motion (SAM) underwent transcatheter aortic valve implantation (TAVI). The invasive peak-to-peak gradient fell from 64 to 7 mmHg, but echocardiography immediately afterwards showed new SAM and severe dynamic left-ventricular-outflow-tract (LVOT) obstruction (170/67 mmHg) with hypotension. Treatment was load-directed (volume expansion, avoidance of vasodilators and inotropes, beta-blockade), with a cardiac myosin inhibitor as an adjunct. Over 4 months the resting LVOT gradient fell to 22/13 mmHg and the provoked peak to 29 mmHg, with SAM no longer seen and preserved ejection fraction.

**Discussion:**

Recognizing this load-dependent obstruction during the procedure and restoring loading, rather than escalating inotropes, is the key lesson.

Learning points

- Relief of the fixed valvular afterload by TAVI can unmask severe dynamic left-ventricular outflow-tract obstruction with systolic anterior motion even when septal thickening is only mild.
- Treatment is immediate and load-directed: restore preload, avoid vasodilators and inotropes, and reduce hypercontractility with beta-blockade.

## Introduction

Transcatheter aortic valve implantation (TAVI) is established treatment for severe calcific aortic stenosis.^1^ Chronic pressure overload produces concentric left ventricular hypertrophy (LVH) that can resemble hypertrophic cardiomyopathy (HCM), and it can also conceal a dynamic outflow-tract obstruction: While the stenotic valve is the dominant fixed obstruction, outflow-tract velocities stay low and systolic anterior motion (SAM) does not develop. Once the valve is relieved, a dynamic SAM-mediated obstruction may appear for the first time, as reported after surgical and transcatheter valve replacement.^2,3^ We describe a man who developed severe dynamic LVOT obstruction with SAM and hypotension immediately after TAVI, managed by restoring loading conditions, with regression over 4 months.

## Summary figure

**Figure:**
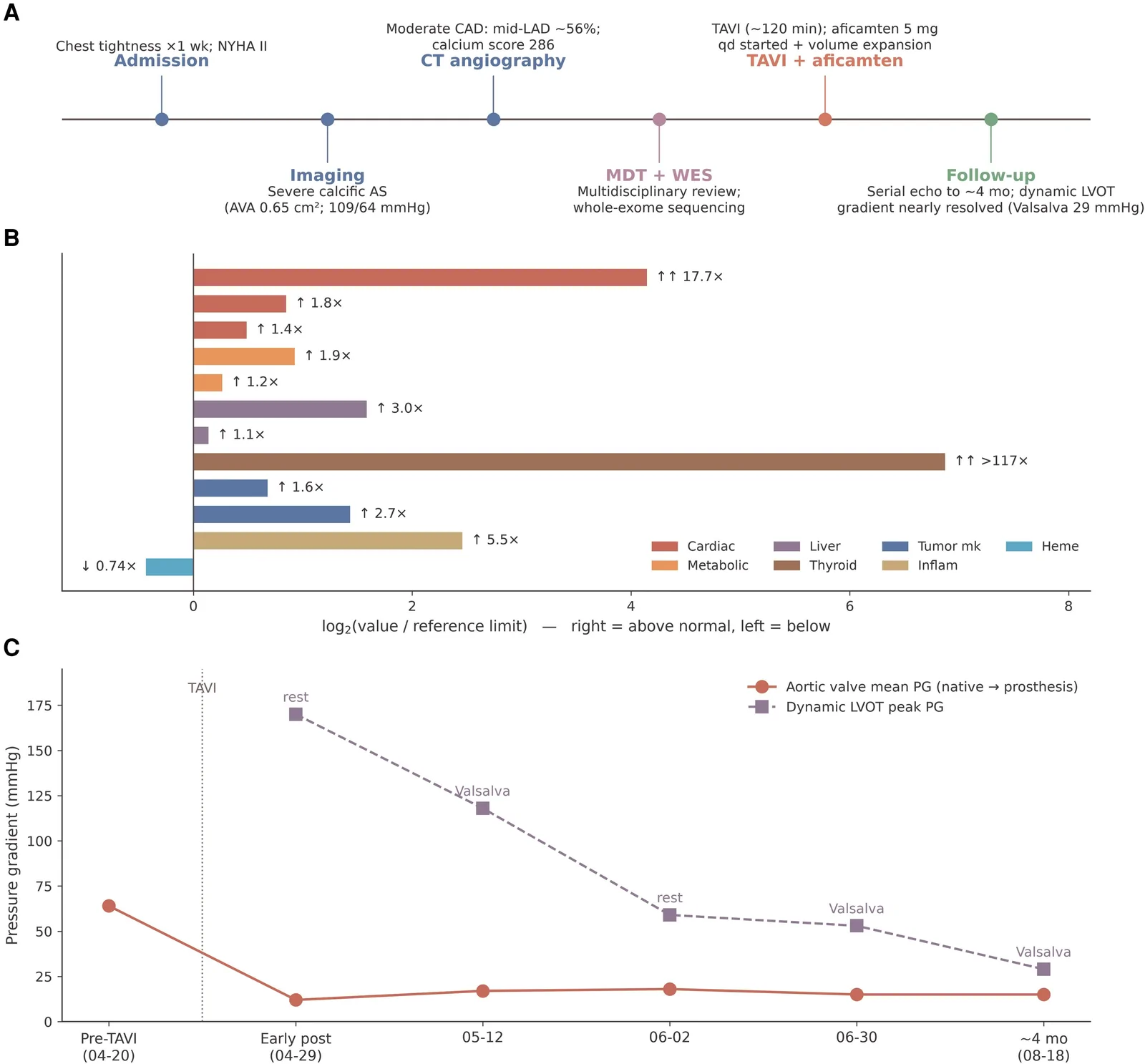
Diagnostic course and haemodynamic evolution. (*A*) Timeline from admission through percutaneous coronary intervention and TAVI to 4-month follow-up. (*B*) Multisystem laboratory abnormalities at presentation, expressed as log_2_ of value divided by the reference limit. (*C*) Serial echocardiography: the fixed aortic-valve gradient falls after TAVI, while a dynamic left-ventricular outflow-tract gradient appears immediately afterwards and regresses over 4 months.

## Case presentation

A 69-year-old man presented with paroxysmal chest tightness (NYHA class II) and an aortic ejection murmur. Transthoracic echocardiography showed severe calcific aortic stenosis (valve area 0.65 cm^2^; peak velocity 5.23 m/s; peak/mean gradient 109/64 mmHg), moderate aortic regurgitation, concentric LV hypertrophy (septum 15 mm, posterior wall 14 mm), preserved ejection fraction (60%), and impaired relaxation (E/e′ 16.9). There was no resting outflow gradient and no SAM before intervention. Coronary CT angiography showed a moderate mid-left-anterior-descending (LAD) lesion (calcium score 286). Comorbidities included autoimmune cirrhosis, Hashimoto thyroiditis, newly diagnosed diabetes, and mild anaemia.

After multidisciplinary review the patient underwent same-session percutaneous coronary intervention (PCI) and TAVI. A drug-eluting stent was implanted in the LAD (final TIMI grade 3 flow), followed by a 29-mm self-expanding transcatheter valve. Invasive pressures before deployment were 242/34 mmHg (LV) and 178/68 mmHg (aorta), a peak-to-peak difference of 64 mmHg; after deployment, they were 218/30 and 211/84 mmHg (difference 7 mmHg), confirming relief of the fixed obstruction.

Echocardiography immediately after deployment showed a normally functioning bioprosthesis (30/12 mmHg) but new SAM of the mitral valve and severe dynamic LVOT obstruction (170/67 mmHg) with hypotension (*Table 1*). Treatment addressed both determinants of the gradient: intravascular volume was expanded to restore preload, vasodilators and inotropes were avoided, and hypercontractility was reduced, first with the cardiac myosin inhibitor aficamten 5 mg once daily, started once the obstruction was recognized, then with metoprolol succinate. The hypotension resolved.

**Table 1:** Timeline

Time	Event
Admission	Chest tightness (NYHA II); severe aortic stenosis on echocardiography (valve area 0.65 cm^2^; 109/64 mmHg); no SAM
Pre-procedure	CT angiography (moderate mid-LAD lesion); multidisciplinary review
Day 0 (TAVI)	Same-session PCI to LAD, then 29-mm self-expanding TAVI; invasive peak-to-peak gradient 64 → 7 mmHg
Immediately post-TAVI	New SAM and severe dynamic LVOT obstruction (170/67 mmHg) with hypotension; load-directed treatment and aficamten started
10 weeks	Resting LVOT gradient 30/15 mmHg; ejection fraction 71%
4 months	Resting LVOT gradient 22/13 mmHg; provoked peak 29 mmHg; no SAM; patient well

Serial echocardiography (*Table 1*) showed progressive regression of the resting LVOT gradient (60/29 mmHg, then 59/26 and 30/15 mmHg over 10 weeks), with ejection fraction rising to 71%. At 4 months (18 August 2026), the patient remained well on the same regimen: resting LVOT gradient 22/13 mmHg, provoked (Valsalva) peak 29 mmHg, no SAM described, mild mitral regurgitation, ejection fraction 65%, and a normally functioning bioprosthesis (25/15 mmHg); the anaemia had resolved and glycaemic control had improved. The recovery reflects the combined effect of volume restoration, beta-blockade, the myosin inhibitor, normalizing loading conditions and reverse remodelling after TAVI.

Whole-exome sequencing during the admission is summarized in the Supplementary material. No reportable variant was found in a well-covered 35-gene HCM panel, favouring a pressure-overload substrate over primary sarcomeric HCM; an incidental *CACNA1S* frameshift was reassessed as of uncertain significance; and a *CYP2C19* *2/*2 poor-metabolizer genotype prompted a recommendation to replace clopidogrel with ticagrelor after individualized risk assessment.^4^

## Discussion

A mildly hypertrophied ventricle without pre-procedural SAM that develops severe dynamic obstruction immediately after TAVI follows from the abrupt removal of a fixed afterload. Before TAVI the stenotic valve is the dominant fixed obstruction; the ventricle ejects slowly through the narrowed valve, outflow-tract velocities stay low, and SAM does not occur. TAVI removes the fixed afterload in one step, and the same hypertrophied, hypercontractile ventricle then empties rapidly into a low-impedance aorta. In a ventricle with a small cavity, a prominent basal septum, preserved-to-supranormal ejection fraction and (here) moderate aortic regurgitation adding flow, the resulting high early-systolic velocities draw the anterior mitral leaflet towards the septum, producing SAM and a dynamic subaortic gradient. Peri-procedural hypovolaemia, tachycardia, and any vasodilators or inotropes make it worse. The determinant is the interaction of ventricular geometry and hypercontractility with a sudden loading change, not the absolute wall thickness, which is why a 15-mm septum can support a resting gradient of 170 mmHg once the valve is open. The same mechanism is reported after surgical aortic-valve replacement^2^ and when obstructive hypertrophy coexists with aortic stenosis.^3^

The mechanism also explains the course. Load-directed treatment targets the dynamic gradient directly, and the hypotension resolved once loading was corrected. Over the following weeks, normalizing loading conditions and reverse remodelling after TAVI reduce the substrate,^5^ in keeping with the fall in resting gradient and the preserved ejection fraction. Reading the hypotension as a load-dependent obstruction rather than prosthesis dysfunction or simple hypovolaemia is what guides correct treatment during the procedure.

The myosin inhibitor was added because the obstruction was severe and hypercontractility was modifiable; these agents are effective in chronic symptomatic obstructive HCM.^6,7^ There are no controlled data for their acute or peri-procedural use, and the drug was an adjunct started after recognition rather than a planned intra-operative agent. Its specific contribution cannot be separated from volume restoration, beta-blockade and reverse remodelling, all acting concurrently, and the ejection fraction rose during follow-up as expected from afterload relief; the observation is therefore hypothesis-generating, with standard load-directed measures remaining the mainstay. Aficamten's disposition is largely independent of the CYP2C19-dependent variability reported for mavacamten,^8^ which is relevant in a poor metabolizer but not in itself a reason for acute use. Genetic testing supported rather than drove management: it was interpreted by category,^9^ the negative HCM panel informing family counselling rather than the phenotype,^10,11^ and the incidental *CACNA1S* variant reclassified with mechanism-aware caution.^12,13^

## Conclusion

Relief of a fixed valvular afterload by TAVI can unmask a severe, load-dependent dynamic LVOT obstruction with SAM in a hyperdynamic, mildly hypertrophied ventricle. It is recognized at the time of deployment and treated by restoring loading, with cardiac myosin inhibition at most an unproven adjunct.

## Lead author biography


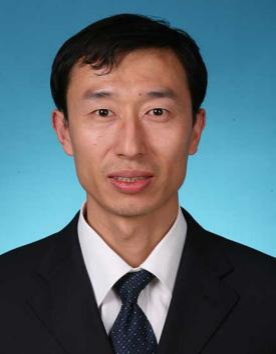
Ruijian Li, Medical Doctor, Chief Physician, Doctoral Supervisor, department of cardiology’s director. He devoted to the treatment and interventional techniques of cardiac critical care and structural heart diseases. With more than 20 years of professional experience in coronary intervention therapy, specializes in complex and high-risk coronary interventional surgery, interventional treatment of structural heart diseases, and other related minimally invasive cardiac procedures. Ruijian Li served as a mentor for coronary intervention training at the National Coronary Training Base, and visiting scholar at Harvard Medical School, Cleveland Heart Center and Hartford Hospital in the United States.

## Supplementary Material

ytag755_Supplementary_Data

## Data Availability

The data are available from the corresponding author on reasonable request; sequencing data are not public owing to privacy restrictions.
