# Supplementary material for: Severe dynamic left-ventricular outflow-tract obstruction unmasked by transcatheter aortic valve implantation: a case report
Source: Eur Heart J Case Rep. 2026 Oct 14;10(10):ytag755. doi: 10.1093/ehjcr/ytag755 (PMC13696958; doi:10.1093/ehjcr/ytag755)
Supplement: Supplementary file 1 [file ytag755_supplementary_data.zip › 09_Supplementary_material.docx]

**Supplementary material**

**Supplementary Figure S1.** Independent read-level reanalysis of the *CACNA1S* and *CYP2C19* loci and sarcomeric-gene coverage.

**Supplementary Figure S2.** *CYP2C19*-guided antiplatelet decision.

**Supplementary Table S1.** Independent read-level confirmation of key variants and on-target depth.

| **Gene** | **Variant** | **rsID** | **hg19** | **Reported** | **Depth** | **Alt fraction** | **Self-called call** |
| --- | --- | --- | --- | --- | --- | --- | --- |
| *CACNA1S* | c.4860dup (p.Val1621ArgfsTer12) | rs761214441 | chr1:201012596 | Heterozygous | 177× | 0.41 | Heterozygous (+1G); Sanger+ |
| *CYP2C19* | c.681G>A (*2) | rs4244285 | chr10:96541616 | Homozygous | 129× | 1.00 | Homozygous *2 |
| *CYP2C19* | c.636G>A (*3) | rs4986893 | chr10:96540410 | Absent | 122× | 0.00 | Reference |
| *CYP2C19* | c.-806C>T (*17) | rs12248560 | chr10:96521657 | Absent | 44× | 0.00 | Reference |

*Sarcomeric HCM-panel mean depth: MYBPC3 333×, TNNI3 221×, MYH7 209×, TPM1 130×, TNNT2 44×.*
